# Supplementary material for: Exosomal microRNAs from Longitudinal Liquid Biopsies for the Prediction of Response to Induction Chemotherapy in High-Risk Neuroblastoma Patients: A Proof of Concept SIOPEN Study ‖
Source: Cancers (Basel). 2019 Sep 30;11(10):1476. doi: 10.3390/cancers11101476 (PMC6826693; doi:10.3390/cancers11101476)
Supplement: Supplementary file 1 [file cancers-11-01476-s001.zip › Figure S1_revised.pdf]

A)

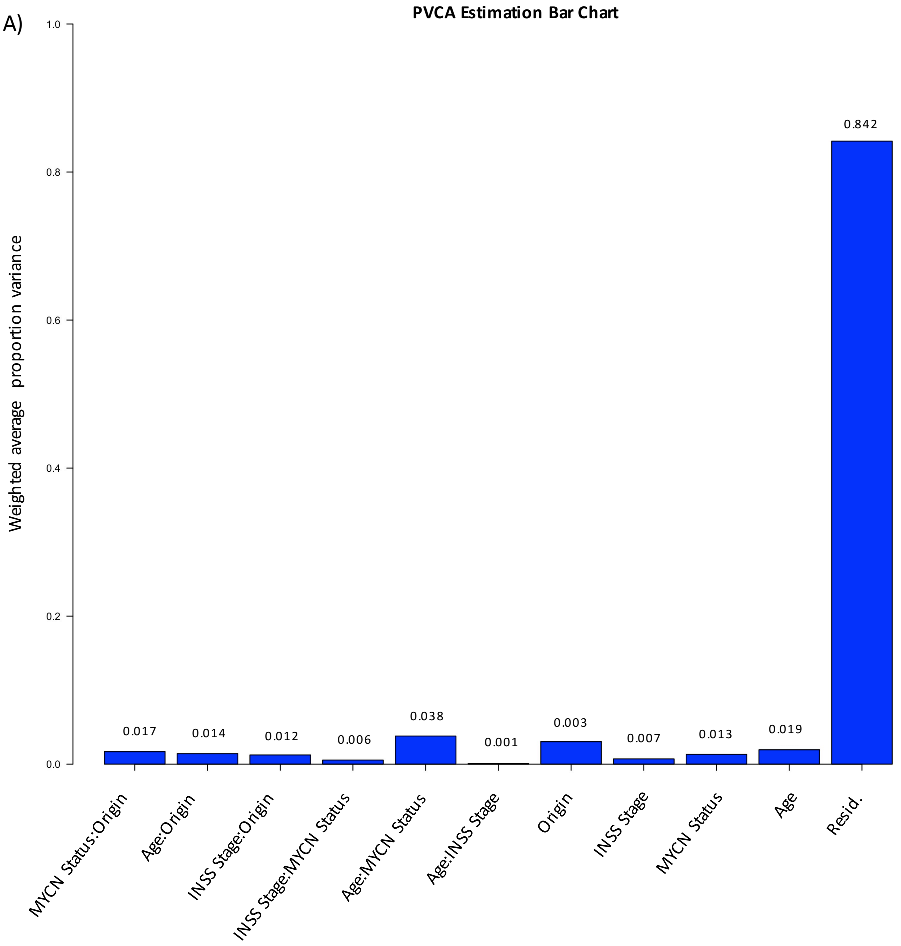

B)

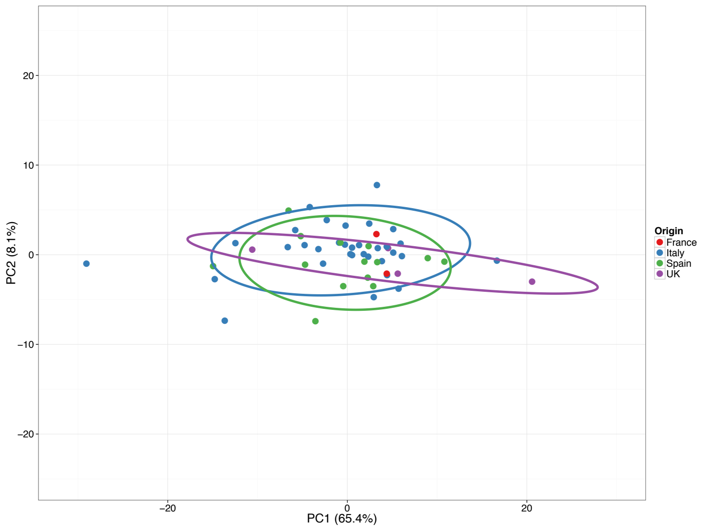

Figure S1. *Origin Batch Effect Evaluation*. (A) Principal Variance Component Analysis Bar Chart showing the impact of different variables (X axis) in introducing technical variability (Y axis) in exo-miR expression evaluation. (B) PCA showing the projection of exo-miR expression profiles associated to the origin of plasma samples (represented by different colours). The resulting clusters are overlapping, confirming that the origin does not significantly influence exo-miR expression profiles. PC=principal component.
