# Supplementary material for: Exosomal microRNAs from Longitudinal Liquid Biopsies for the Prediction of Response to Induction Chemotherapy in High-Risk Neuroblastoma Patients: A Proof of Concept SIOPEN Study ‖
Source: Cancers (Basel). 2019 Sep 30;11(10):1476. doi: 10.3390/cancers11101476 (PMC6826693; doi:10.3390/cancers11101476)
Supplement: Supplementary file 1 [file cancers-11-01476-s001.zip › Figure S2.pdf]

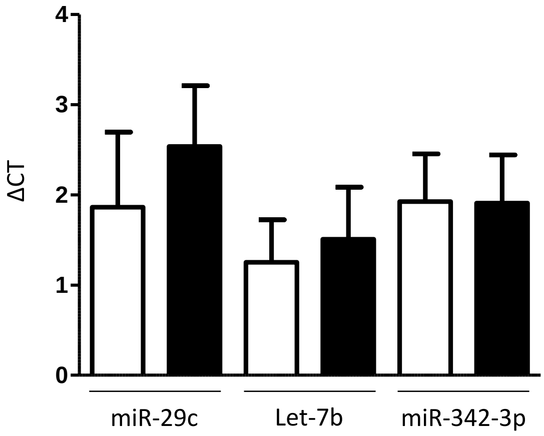

Figure S2. *Exo-miR signature validation by Real-Time PCR (RT-PCR)*. The expression of miR-29c, miR-342-3p, and let-7b was validated by RTqPCR. The bar charts show the differences of CT values after the induction chemotherapy measured by both Array Card (black bars) and RTqPCR (white bars). The difference between the ΔCT values measured with both techniques is not significant (T-test p value > 0.05).
