# Supplementary material for: Exosomal microRNAs from Longitudinal Liquid Biopsies for the Prediction of Response to Induction Chemotherapy in High-Risk Neuroblastoma Patients: A Proof of Concept SIOPEN Study ‖
Source: Cancers (Basel). 2019 Sep 30;11(10):1476. doi: 10.3390/cancers11101476 (PMC6826693; doi:10.3390/cancers11101476)
Supplement: Supplementary file 1 [file cancers-11-01476-s001.zip › Figure S3.pdf]

A)

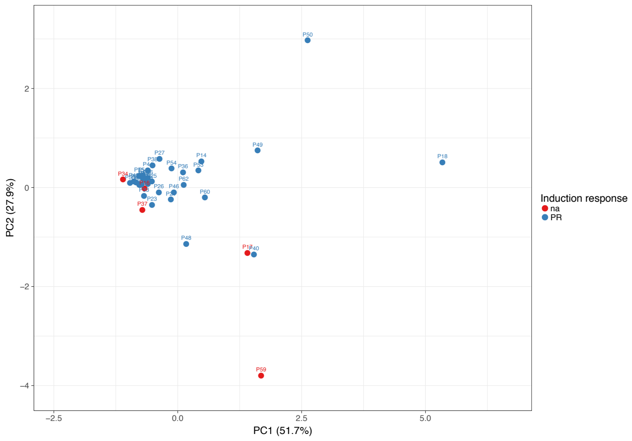


B)

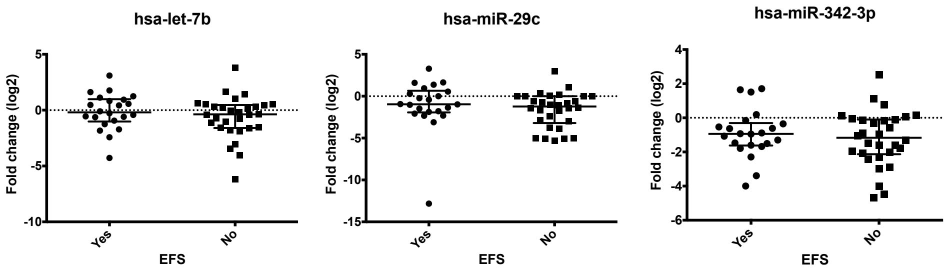


***Figure S3****. Exo-miRNA signature in partial response patients and its efficacy in predicting Event Free Survival (EFS).* (A) The PCA analysis showed that PR patients could not be differentiated into two different groups of response on the basis of the 3 exo-miRNAs expression. PC= principal component, PR = partial response, na= induction chemotherapy response not available. (B) The dotplot graphs show the fold change values of each exo-miRNA between patients with a poor prognosis (EFS=yes) and patients with a good prognosis (EFS=no). The median and the interquantile range values are reported. According to the association analysis, the 3 exo-miRNA signature was not able not effectively predict EFS (Mann–Whitney test p value >0.05).
