# Supplementary material for: Exosomal microRNAs from Longitudinal Liquid Biopsies for the Prediction of Response to Induction Chemotherapy in High-Risk Neuroblastoma Patients: A Proof of Concept SIOPEN Study ‖
Source: Cancers (Basel). 2019 Sep 30;11(10):1476. doi: 10.3390/cancers11101476 (PMC6826693; doi:10.3390/cancers11101476)
Supplement: Supplementary file 1 [file cancers-11-01476-s001.zip › Figure S4.pdf]

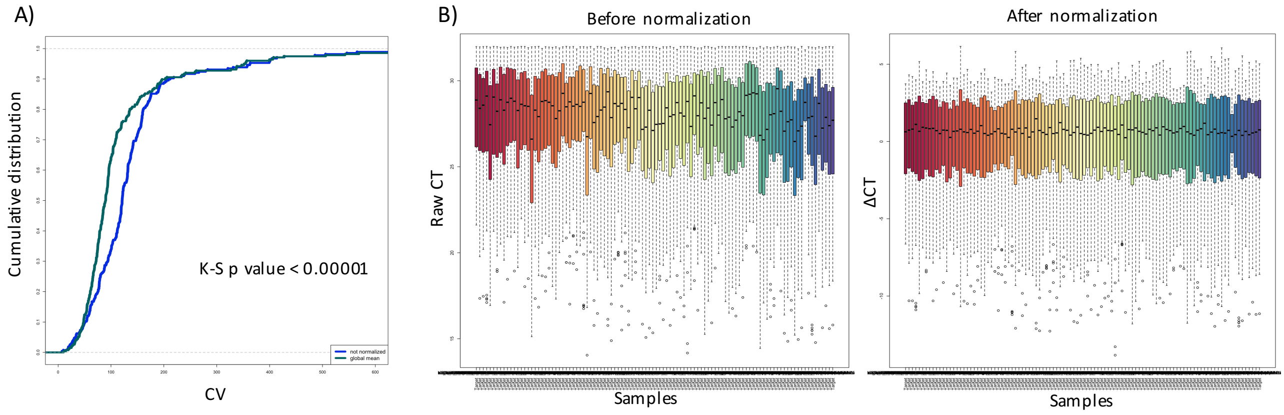

Figure S4. *Global Mean Normalization effectively reduces technical variability.* (A) Cumulative distribution of miR coefficient of variation (CV) values before (blue curve) and after (green curve) normalization. The p value is calculated by Kolmogorov-Smirnov test. (B) Boxplot showing the distribution of CT values before normalization and the distribution of normalized values (ΔCT) in the cohort of HR-NB patients.
